# Fatal re-expansion of hypertensive cerebellar hematoma

**DOI:** 10.11604/pamj.2014.18.122.4114

**Published:** 2014-06-07

**Authors:** Ali Akhaddar, El Mehdi Atmane

**Affiliations:** 1 Department of Neurosurgery, Avicenne Military Hospital, Marrakech, Morocco; 2 University of Mohammed V Souissi, Rabat, Morocco; 3 Department of Radiology, Avicenne Military Hospital, Marrakech, Morocco

**Keywords:** Cerebellar hematoma, hypertension, intracerebral hemorrhage

## Image in medicine

Intracerebral hemorrhage is one of the most devastating forms of stroke especially in the posterior fossa. Expansion of cerebral hematomas was common in the acute phase (< 6 hours) but rarely reported 24 hours later. A 70-year-old man, with history of hypertension, was admitted to the emergency service with the complaint of headache, dizziness and vomiting. He was neurologically intact except for neck stiffness. A cranial CT-scan demonstrated a left hemispheric cerebellar hematoma (about 2 cm in diameter) and a fourth ventricular hemorrhage without hydrocephalus (A). The patient was kept under observation. About 32 hours of onset, the patient complained of severe occipital headache and sudden loss of consciousness. He was brought to the ICU, where he appeared drowsy and bradipnoic. Neurological examination showed anisocoria. An emergency CT-scan revealed an increase of hematoma volume, the diameter was about three times as large with acute hydrocephalus (B). The patient was transferred to the operating room but unfortunately died before performing any surgery. This case shows that we should always consider the risk of hematomas enlargement or re-expansion following a hemorrhagic stroke. This secondary phenomenon can occur late and may be cause a rapid fatal outcome if not detected and managed early.

**Figure 1:**
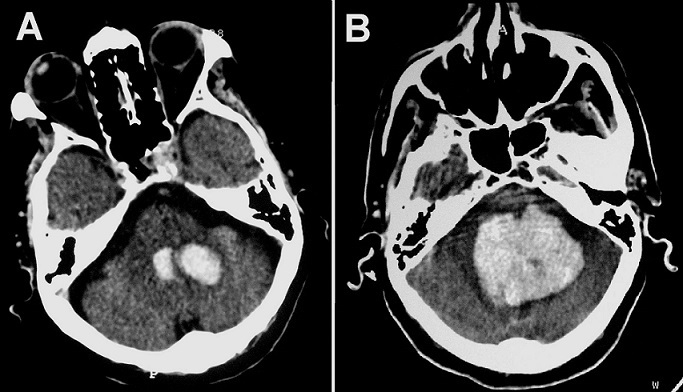
(A): Initial cranial CT-scan showing an acute left hemispheric cerebellar hematoma (about 2 cm in diameter) and a fourth ventricular hemorrhage. (B): Control CT-scan performing about 32 hours of onset revealing an important increase of hematoma volume: diameter about three times as large

